# Comment on "Effectiveness and safety of stem cell therapy for diabetic foot: a meta-analysis update"

**DOI:** 10.1186/s13287-023-03608-w

**Published:** 2024-03-21

**Authors:** Yan Bai, Fan Zhang

**Affiliations:** 1 Department of Cardiology, Longhua Hospital Shanghai University of Traditional Chinese Medicine, Shanghai, China; 2 Department of Nephrology A, Longhua Hospital Shanghai University of Traditional Chinese Medicine, Shanghai, China

**Keywords:** Stem cell, Diabetic foot, Systematic review, Meta-analysis

## Abstract

In the study published by Sun et al., a systematic review and meta-analysis illustrated the advantageous of stem cell therapy in diabetic foot and can improve the quality of life of patients. Nevertheless, the authors had a lack of knowledge regarding the methodology of the meta-analysis, which had four main aspects: (1) The textual report is inconsistent with the forest plot results, i.e., the authors have insufficient knowledge of RevMan. (2) The "zero event" needs to be corrected for summary analysis. (3) Lack of aesthetics in the forest plots. (4) Registration is recommended for systematic reviews and meta-analyses.

## Letter to the editor:

We read the recent article published by Sun et al. [1], which specifies from a meta-analysis that stem cells are significantly more effective than traditional methods in treating diabetic foot and can improve patient's quality of life after treatment. We congratulate Sun et al. for their results, but several methodological issues with the article need to be highlighted so as not to mislead the readers.

First, a fatal error lies in the authors' lack of awareness about the Revman software. Taking *Fig. 3* as an example, the summary diamond falls on the "Control[control]" side, so the result of the forest plot should be that "the healing rate of ulcers or wounds in the control group was higher than in the cell-treated group", but the authors report the opposite. In fact, when using Revman's forest plot, we need to consider whether the outcome is a "favorable event" or an "unfavorable event" [2]. Obviously, the healing rate of ulcers or wounds is a "favorable event," and the label of the forest plot needs to be revised.

Second, as suggested by Friedrich et al. [3], when incorporating a "zero event" trial, the constant continuity correction method should be applied by adding a correction factor of 0.5 in case of zero events in one group. As we know from the forest plot in the text, the authors have not corrected it.

Third, although it is not a methodological error, we argue that the authors should have tried to be as aesthetically pleasing as possible when drawing the forest plots. For example, in *Fig. 6*, the reader cannot even be notified of the summarized diamond; in *Fig. 8*, the diamond appears broken. It is reasonable to assume that Stem Cell Research & Therapy is a high-quality journal [4], but editors can avoid such phenomena when dealing with similar manuscripts. The forest plots can be visualized more in the Revman software by dragging the scroll bar.

Fourth, study protocols help to improve the transparency of the review methodology and avoid bias in the reporting of results [5]. We recommend that the authors register with the International Prospective Register of Systematic Reviews (PROSPERO) or the International Platform of Registered Systematic Review and Meta-analysis Protocols (INPLASY) before conducting a systematic review and meta-analysis.

Figures 3, 6, and 8 mentioned in this paper are not the figures of this letter, but the figures of the published paper of Sun et al. (Effectiveness and safety of stem cell therapy for diabetic foot: a meta-analysis update).

## Data Availability

Not applicable.
